# Clients’ expectations and experiences with providers of menstrual regulation: a qualitative study in Bangladesh

**DOI:** 10.1186/s12905-024-03137-5

**Published:** 2024-05-16

**Authors:** Ana Maria Ramirez, Tanzila Tabassum, Sofia Filippa, Anna Katz, Rezwana Chowdhury, Chiara Bercu, Sarah E. Baum

**Affiliations:** 1 Ibis Reproductive Health, Oakland, CA USA; 2 Terre des Hommes, Dhaka, Bangladesh; 3 Ipas Bangladesh, Dhaka, Bangladesh

**Keywords:** Menstrual regulation, Quality of care, Abortion stigma, Bangladesh, Person-centered care

## Abstract

**Background:**

Menstrual Regulation (MR) has been legal in Bangladesh since 1979 in an effort to reduce maternal mortality from unsafe abortion care. However, access to high-quality and patient-centered MR care remains a challenge. This analysis aimed to explore what clients know before going into care and the experience itself across a variety of service delivery sites where MR care is available.

**Methods:**

We conducted 26 qualitative semi-structured interviews with MR clients who were recruited from three different service delivery sites in Dhaka, Bangladesh from January to March 2019. Interviews explored client expectations and beliefs about MR care, the experience of the care they received, and their perception of the quality of that care. We conducted a thematic content analysis using a priori and emergent codes.

**Results:**

Clients overall lacked knowledge about MR care and held fears about the damage to their bodies after receiving care. Despite their fears, roughly half the clients held positive expectations about the care they would receive. Call center clients felt the most prepared by their provider about what to expect during their MR care. During counseling sessions, providers at in-facility locations reinforced the perception of risk of future fertility as a result of MR and commonly questioned clients on their need for MR services. Some even attempted to dissuade nulliparous women from getting the care. Clients received this type of questioning throughout their time at the facilities, not just from their medical providers. The majority of clients perceived their care as good and rationalized these comments from their providers as coming from a caring place. However, a handful of clients did report bad care and negative feelings about their interactions with providers and other clinical staff.

**Conclusion:**

Providers and clinical staff can play a key role in shaping the experience of clients accessing MR care. Training on accurate knowledge about the safety and effectiveness of MR, and the importance of client communication could help improve client knowledge and person-centered quality of MR care.

**Supplementary Information:**

The online version contains supplementary material available at 10.1186/s12905-024-03137-5.

## Background

Abortion in Bangladesh is illegal except to save the life of the mother [1]. Menstrual regulation (MR) defined as a “procedure of regulating the menstrual cycle when menstruation is absent for a short duration” was approved in 1979 to address high maternal morbidity and mortality rates as a result of unsafe abortion [2]. MR services include manual vacuum aspiration (MVA) or a combination of mifepristone and misoprostol, which was approved in 2013. As of 2014, 88% of MR services were performed using MVA, while 11% were done using the medication regimen [3]; however, more recent country-level data is needed to better characterize the current rates at which each MR method is performed. Bangladeshi women^1^ can access MR care from several service delivery sites, including government hospitals, clinics operated by non-governmental organizations (NGOs), and pharmacies that sell MR pills. The information provided to the person seeking MR varies across these service delivery sites and research suggests that pharmacists often offer inconsistent information about how to take the pills and what to expect [4]. More recently, call centers have been established to inform callers where to access and how to safely take MR pills [5]. In order to increase access to MR, the Bangladeshi government implemented trainings with mid-level providers, such as family welfare visitors who also provide family planning services and created guidelines to improve quality of service provision [2].

Knowledge and use of MR services among Bangladeshi women has fluctuated over time. From 2001 to 2014, knowledge of MR decreased approximately by half, with only 45% of ever-married Bangladeshi women reporting that they had ever heard of MR in 2014 [6], while the prevalence and incidence of MR procedures decreased [3, 6]. More recent country-level data shows that 71% of ever-married know about MR, and the overall prevalence of MR has increased [7], thus suggesting a potential association between knowledge of MR and use of MR services. Access to and awareness of MR services has also been associated with geographic location and economic status, with women of higher socio-economic status and those living in urban areas more likely to access MR services [6].

While a lack of overall awareness of MR, including its legal status, gestational age cutoffs, and where or how to access it, presents one substantial barrier to accessing high-quality safe MR services [8], there is also stigma around MR services that present additional barriers. While there is no legal restriction for unmarried women to access services, there is substantial social stigma for unmarried women who get pregnant and an assumption that only married women would need to access MR services [9]. Some providers reinforce this stigma, charging unmarried women more and requiring consent from other family members such as spouses or parents when there is no legal requirement to do so, and even deny unmarried women MR services [9–12]. Given the loss of privacy that results from requiring family member consent, some women may avoid seeking MR care all together.

MR service delivery in Bangladesh has been studied primarily quantitatively [13–16], with few qualitative studies focusing on incorporating MR clients’ perspectives on the quality of their care. One qualitative study among 10 MR clients in Dhaka found that providers financially exploited clients by charging more to unmarried clients or clients further along in their pregnancy [10]. Another qualitative study that specifically recruited women who were initially turned away from care found that clients’ perception of quality of MR care focused on the cleanliness of a facility and experience with staff; though it was not clear how much either of these characteristics mattered so long as the MR procedure was completed without complication [8].

In order to better understand the MR client perspective on quality of care, with a focus on interpersonal interactions, this exploratory study aimed to assess the knowledge and expectations clients had prior to their MR care and explore experiences with providers during the MR process.

## Methods

Between January and March 2019, TT and SM conducted semi-structured in-depth interviews with women who received MR services in Dhaka, Bangladesh as part of a larger study. The larger study, the results of which were published in September 2023 [17], sought to understand client priorities in abortion quality of care in four legally diverse countries: Argentina, Bangladesh, Ethiopia, and Nigeria. Clients were recruited from a diversity of service delivery sites in each country; in Bangladesh clients were recruited at three service-delivery sites, including at two NGO sites (NGO 1 and NGO 2) and a government maternity hospital. At NGO 1, clients were recruited at the organization’s clinic, while at NGO 2 clients were recruited through its call center. To be eligible for the study, women had to be 15 years or older, have received MR services within the past three months, and be able to provide consent to participate in the study.

AR, AK, CB, SF and SB developed an initial interview guide for the larger multi-country study, and then edited it based on feedback from TT and RZ in Bangladesh to ensure that questions were contextually relevant to the service delivery sites and Bangladeshi culture. Interview topics included perception of good and bad quality medical care, community perceptions of MR, knowledge and expectations of MR prior to services, and experience with MR services (Supplementary File I).

Recruitment sites were purposively selected to ensure a range of service delivery sites were represented within Dhaka and based on existing partnerships with service providers. The hospital and NGO 1’s clinic provided both medication and procedural MR. Procedural MR clients at both facilities are offered local and general anesthesia for additional costs and could have their services completed on the same day as their ultrasound. MR with medication (MRM) clients complete their ultrasound in-facility and are then provided pills to take at home to complete their MR; clients are asked to return to the facilities for a follow-up ultrasound and contraceptive counseling. NGO 2’s call center is affiliated with a clinic ran by NGO 2. The call center is staffed 24/7 by trained mid-level medical service providers who provide clients with information and counseling about where to access the two types of MR services in Bangladesh, refer clients to NGO 2’s clinic or other health facilities, describe the MRM process, including dosage and side effects for those already in possession of MR pills from pharmacies or NGO 2’s clinic, and do follow-up counseling for those who have completed MR in both in and out of clinic contexts. Callers are able to contact the call center at any time during their MR process with any questions. The call center recruited callers who contacted them with questions about in-clinic MRM and procedural MR services, as well as callers who had MRM outside of clinic settings by sourcing pills from pharmacies. Clients recruited from the government maternity hospital and NGO 1’s clinic were recruited in-person by TT and SM after completion of their MR services, that is, after post-procedure counseling during the client’s visit and/or after their follow-up visit. Interviews were conducted in-person in a private space within the facility. Eligible clients who contacted NGO 2’s call center for the first time or during follow-up calls were asked by call center staff if they would be interested in participating in an in-depth interview about their MR service. Interested clients then provided consent to share their personal phone numbers with TT and SM who formally recruited callers into the study. Due to concerns about client privacy and security, only clients with personal phones were asked to participate in in-depth interviews and not those who shared phones with others. All call center clients were interviewed over the phone at a time of their choosing after their MR service was completed. No participant dropped out of the study after enrolling. The study team decided to stop recruitment once thematic saturation was reached. All interviews were conducted in Bangla, lasted between 30 and 60 min, and were audio recorded; participants received 500 Bangladeshi taka (approx. $5–6 USD) for their time via online mobile transaction. Field notes were done after each interview.

Interviews were transcribed in Bangla and then translated to English. AR, AK, CB, SF and SB developed an initial codebook used for the larger study based on a priori quality of care domains and themes that emerged from the data. They then applied the codes independently to four transcripts of the larger data set. After the team met to discuss the discrepancies and gaps, they collapsed and clarified code definitions; SF and CB then coded all the transcripts of Bangladeshi participants using the final codebook on MAXQDA, Verbi Software. The framework for this analysis relied on the Person-Centered Care Frameworks for Reproductive Equity [18], and quality domains that emerged from other qualitative research done with abortion clients [19–21]. In particular, the Person-Centered Framework for Reproductive Equity helped guide the development of codes focused on capturing people’s previous experiences with care, previous abortion knowledge and expectations of care, as well as codes to document participants’ care experiences according to different person-centered outcomes. We conducted thematic analysis of emerging patterns and themes among the entire sample in Bangladesh and present findings with illustrative quotes, which are identified by age, service delivery site(s) that the participant interacted with, MR context (in vs. out of clinic), and MR method.

This study was carried out in accordance with the ethical principles of the Declaration of Helsinki. All study team members, who all identified as women, are trained in conducting ethical research. All participants were informed of the study topics and aims and consented prior to participating in the interviews. Participants were aware of the voluntary nature of the study and were able to withdraw at any time. Participants from the in-facility sites were asked to provide their written informed consent, while participants from the call center were asked to provide their verbal consent [22]. This study was approved by Allendale Investigational Review Board (USA) (Protocol Number ASQ092018), Marie Stopes International Ethics Review Committee (UK) (Protocol Number 023 − 18), and the Bangladesh Medical Research Counsel (Bangladesh) (Protocol Number 164 13 11 2018). We report our Methods and Results in accordance with COREQ checklist for qualitative research (Supplementary File II).

## Results

We interviewed 26 MR clients across the three recruitment sites, 16 clients from NGO 2’s call center, five clients from the maternity hospital, and five clients from NGO 1’s clinic. All but one client recruited from the maternity hospital and one client from NGOs 1’s clinic received procedural MR services and roughly half of them received some local or general anesthesia during their care. Of the clients recruited from NGO 2’s call center, eleven had MRM, seven of which accessed care outside of a clinic at places such as pharmacies. The rest of the participants recruited from the call center received procedural MR or MRM services from NGO 2’s clinic in addition to contacting the call center for support and follow-up. Parity varied across the sample with five nulliparous clients and the rest with a range of 1–5 children. Table 1 details additional demographic characteristics of clients across service delivery sites.

**Table 1:** Participant Characteristics

Age	*n* (%)
≤ 18 years old	2 (8%)
19–24 years old	10 (38%)
25–30 years old	9 (35%)
≥ 31 years old	5 (19%)
**Marital status**	
Not Married Married	0 (0%) 26 (100%)
**Employment**	
Employed	8 (31%)
Student	7 (27%)
Unemployed	10 (38%)
Unknown	1 (4%)
**MR method**	
Procedural (MVA)	14 (54%)
Medication	12 (46%)
**Gestational age**	
≤ 7 weeks	21 (81%)
8–10 weeks	3 (11%)
11–12 weeks	2 (8%)
**Recruitment site**	
Government Maternity Hospital	5 (19%)
NGO 1 Clinic	5 (19%)
NGO 2 Call Center	16 (62%)
**Prior MR**	
Yes	6 (23%)
No	20 (77%)

### Prior knowledge and expectations

#### Lack of general knowledge and fearful expectations of MR

When asked to report their knowledge about MR prior to accessing care, few clients reported knowing about the procedural process or about the option for medication use. This was even true for the clients who had had previous MR services, either because they had been under anesthesia for their previous MR or they had had a different type of MR than the one they ended up obtaining through one of the recruitment sites of this study. Clients commonly held fears about potential negative consequences from MR based on communication with close female relatives or others in their local community. The most commonly held fears were that MR could lead to future infertility or damage to the uterus or cancer as a result of “dirty instruments”. Clients were also concerned about pain during the procedure and damage to the body caused by infections, especially if a woman had more than one MR, framed as having too many MRs. For instance, one client shared,

What I mean is, they [people] say that if you do it too much, then you can have complications in that area. And then later you will often discover that you might get cancer in your womb. Because you see, if you have this done over and over again, then it becomes like an infection/scarring of that place, doesn’t it? *(26 years old, NGO 1 Clinic, in-clinic MRM)*

Another client who received pills from a pharmacy and then contacted the call center for support also expressed fears related to MR,

I have heard that you can have a lot of problems [as a result of this procedure]. Many people don’t have children. Or if they have one child, then there might be a long time during which they are unable to get pregnant. (*28 years old, NGO 2 Call Center, out-of-clinic MRM*)

The second most common fear women spoke about was a general fear of the MR process itself. This was often a result of not knowing what to expect during the procedure or not feeling informed by their provider going into care. Some clients were able to articulate what they were fearful of, such as the use of instruments or going into what they perceived to be surgery, but many were not and just described feeling fearful generally.

#### Expectations about provider interactions

When asked about how they anticipated providers to treat them before seeking care, approximately half of the participants reported anticipating positive treatment and half expressed negative expectations about provider behavior. Negative expectations arose from fear of judgment from staff in the form of verbal shaming and chastising, as explained by this client,

I had very negative ideas about that. I was very worried about it – that they might say all kinds of things to me. First of all, this is not a good thing to do. We are Muslims, and this is a sinful thing. Because of that, they might well say something bad to me. *(23 years old, Maternity Hospital, in-clinic procedural MR)*

Another client was worried about being perceived as irresponsible by hospital staff for not using contraception to prevent the pregnancy. The client shared,

Yes, I was worried that they might tell me off, saying ‘Why did you not use some kind of contraceptive method before this happened? A stable, long term method [is best].’ I was convinced that since I was going to have an MR done, they would definitely behave badly with me – namely, that they would make some digs at my expense. They do the MR for you, but they still say things like that. ‘Why are you doing this? Did you not take any preventive measures in this regard?’ They are bound to say these kinds of things. (*22 years old, Maternity Hospital, in-clinic procedural MR)*

Similarly, a procedural MR client who was returning for her second MR service felt concerned her providers would judge her. In addition, during her prior abortion, the providers had encouraged her to have tubal ligation but she never did, so she thought that coming back again for MR would upset them. There was an expectation among clients that unmarried women in particular would be treated differently than married women who presented for MR care. Specifically, that unmarried women would be shamed for having socially unacceptable premarital sex and becoming pregnant. Some clients even felt that unmarried women would be turned away from care. Although every client interviewed reported being married, one young client who sought services from NGO 1’s clinic was worried that staff and providers might assume she was unmarried and would be treated as such, as she described,

Yes, I was really very afraid. I thought that because I was relatively young, because my husband had not come with me, they might think that I was a bad woman, that I had fallen pregnant as the result of an illegitimate relationship, and they might say dirty things about me. I was very afraid about whether they would make certain assumptions when they heard that I was having an MR. I thought that they would think that I was lying. (*16 years old, NGO 1 Clinic, in-clinic procedural MR)*

Among clients who held positive expectations of provider and staff treatment, their expectations were based on positive associations with the facility for providing good care. These positive associations were based on the recommendations clients received from those they trusted to seek care from these facilities, while others had previous positive experiences of their own at these facilities, either for MR care specifically or other sexual and reproductive health care. One call center client who had received services at NGO 2’s clinic had positive expectations for MR care because of the NGO 2’s clinic reputation,

No, I had already heard earlier that you people deal with various kinds of problems that women face – like all the private difficulties [we experience], all of those things. And also, that you provide a very good service, and that for this MR procedure, [the clinic] provides very good care. This is something that I already heard beforehand. (*18 years old, NGO 2 Call Center + Clinic, in-clinic MRM)*

For one client, her positive expectations about NGO 2’s clinic were the reason she chose to seek care from that facility,

My expectations were certainly high – I felt that I would be treated well, that there would be a good environment there [at NGO 2’s clinic]. I felt that it would be clean, and that they would undertake the procedure with due care. That is [why] I went to them. (*32 years old, NGO 2 Call Center + Clinic, in-clinic procedural MR*)

### Experience with MR care

#### Provider counseling

Counseling sessions with providers primarily during in-clinic care gave clients the perception that MR care was available if absolutely necessary, but that it is not a good thing to do and should be avoided if possible. Five clients experienced instances where their MR providers or other staff involved in their care attempted to discourage or talk them out of their MR procedure. In some instances, providers framed their objections as questions to the clients asking why they did not want to keep the “baby” or other language that suggested fetal personhood. On one occasion, a provider tried to persuade the client out of their current MR in exchange for a permanent contraceptive method after her pregnancy, indicating that getting an MR service was ultimately a bad thing. “*They said, ‘Your baby is little.’ One of the doctors said, ‘You should keep this baby. After you have had it, you can have an, um [ligation], done.’”* (*24 years old, NGO 1 Clinic, in-clinic procedural MR)*.

Providers specifically made pleas to younger nulliparous women, stating that they should keep the pregnancy because it would be their first child. Four clients described their providers trying to convince them out of having MR specifically because it was their first pregnancy and that they should keep it for that reason alone. One client described how the doctor urged her to discuss her decision to get an MR service with her husband since this was her first pregnancy,

They sent me to have an ultrasound done. And there, I spoke to the doctor. She tried to get me to understand that I should discuss this with my husband, that I should keep this baby, because it was my first child. *(24 years old, NGO 1 Clinic, in-clinic procedural MR)*

Some clients were questioned multiple times by a variety of staff throughout service provision at clinics. A client who received MRM at NGO 1’s clinic had multiple interactions with staff throughout her service who first asked her, “*‘Why don’t you want to have this baby?’”* She then had to explain her reasoning to each of the staff and providers up until she received her service.

In some instances, providers also emphasized the importance of using long-acting reversable contraception as a way to avoid the need for future MR services, again emphasizing that MR can be damaging to the body. When a call center client shared that she had had a previous MR with the providers at NGO 2’s clinic, one provider said, “*It is not right to keep having an MR done over and over again. That is damaging for your health.” (26 years old, NGO 2 Call Center + Clinic, in-clinic procedural MR)*. This same client said that her provider at NGO 2’s clinic was insistent on her receiving a shot or an implant because she sought MR services “too close together”. Despite the client’s fear of inserting any method into her body, the provider only stopped insisting once the client’s husband stepped in and did not give “permission” for these contraception methods.

Clients also commented on the information that providers gave them on what to expect during their MR process. Most clients who contacted NGO 2’s call center and a few from NGO 1’s clinic and maternity hospital felt well informed about what to expect during either their procedural MR or MRM process. MRM clients described receiving information about medication dosage and timing, suggestions on what to eat before taking the pills, and information about side effects. For instance, one client who got the pills from a pharmacist described contacting NGO 2’s call center for information and clarification on how to take them, “*…I was having some difficulty in understanding his [the pharmacist] instructions. So because of that, I called you*.” *(25 years old, NGO 2 Call Center, out-of-clinic MRM).*

For procedural MR clients feeling well informed meant knowing about what to expect during their time at the clinic. However, it was more common for participants who had procedural MR to feel less informed about the steps of the procedural MR process. One client said that staff were in too much of a hurry to provide sufficient information and as a result she felt unprepared for her procedural MR experience,

I asked them again and again, but the truth is, saying something at that point, or understanding what was happening was made more difficult by the fact that they were all in a tremendous hurry and they were working in a kind of [hectic] way. So in that kind of frightening situation, how many times can you ask them about it? *(35 years old, NGO 2 Call Center + Clinic, in-clinic procedural MR)*

In-clinic providers tended to reinforce a misperception of physical risk and safety concerns associated with MR, specifically risk of future infertility. At the maternity hospital clients were provided with a pamphlet before their MR care that overly emphasized and misrepresented the risk of negative potential consequences from procedural MR. Several clients from this facility spoke about reading these negative consequences and one client said that she started feeling bad and uncertain about what could happen as a result of MR. Providers also discussed these risks with clients during counseling sessions when discussing the option of MR. For instance, one participant shared,

She said [the provider] that the uterus might get perforated, and there might be a lot of bleeding, so you might not be able to have children in the future – these are things that the doctor said and they were also written down on the form. (*22 years old, Maternity Hospital, in-clinic procedural MR*)

Not all clients spoke about how providers presented the choice between procedural MR and MRM. At least six were explicit about not having an option or choice presented to them during counseling. All but one of these clients was told they were having a procedural MR. Two clients were told that they were too far along for MRM, and three had providers choose the procedural method for them. One client shared, *“They are the ones who, uh, actually looked at this and decided that which method would be the most appropriate for me.” (32 years old, NGO 1 Clinic, in-clinic procedural MR).*

#### Positive interpersonal interactions between clients and providers

Clients across the different service delivery sites primarily reported positive perceptions of care from their providers and other staff. Positive experiences were described as being spoken to nicely, pleasantly, or politely and providers “behaving well”, sometimes described as the absence of bad care. One client shared, “*But the way that I understood/saw it, everyone there [NGO 2’s clinic] behaved well. They spoke to me nicely. They did everything properly. I did not notice anything bad about it” (22 years old, NGO 2 Call Center + Clinic, in-clinic procedural MR)*. Clients also appreciated when providers reassured them of the safety of the procedure, reminding them that there was nothing to worry about. Clients felt encouraged and supported by these verbal affirmations. One client who had an MRM spoke about how the staff at the call center was able to allay her fears,

They [call center staff] behaved very nicely. That Apu [call center counselor] was really good. She spoke to me very nicely. And she said, ‘There is nothing for you to worry about with respect to this matter.’ She gave me courage. (*28 years old, NGO 2 Call Center, out-of-clinic MRM*)

Despite instances where providers attempted to dissuade clients from seeking MR services, these clients still felt supported during their MR service, describing their service was “quite good”. This discordance was explained by a change in provider treatment once the providers accepted that the client would move forward with her MR; clients felt that after providers agreed to provide MR care they were more encouraging and supportive than during their initial interactions. Clients were also able to rationalize this type of treatment from their providers as either being deserving of this treatment, or that the providers were well intentioned and that clients were spoken to this way for their own benefit. One client who was told to keep her pregnancy by her provider still felt like her provider came from a place of good intention,

No, they told me this – that I am young - because they were thinking of my welfare. I mean, this has been more of a risk for me, because of [the fact that I have] three children already. They said it with the best of intentions. I don’t think that this was a bad thing to say. *(24 years old, NGO 1 Clinic, in-clinic procedural MR)*

Another procedural MR client at the maternity hospital who experienced multiple rounds of questioning by staff about her desire to have MR and presenting around 12 weeks’ gestation said that the scolding she received was warranted for how “late” she was coming for care. She ultimately felt that the care she received was great because providers were kind, patient, explained the procedures, and listened to patients. She also thought that providers did not speak aggressively to her the way they could have and asked for consent before they did anything.

#### Experience with bad care

There were a few instances where clients described feeling that their experiences with staff or providers were bad. Clients defined these negative interactions as providers who were “rude” or short tempered with them. This left some clients feeling upset, hesitant to express concerns, and at times even humiliated by how they were treated. During contraceptive counseling at NGO 2’s clinic, one client said that the provider grew angry and spoke to her in an “unpleasant way” such that it dissuaded the client from asking additional questions she had. The client said, *“I wanted to ask a couple of questions, but after seeing their attitude, I actually decided against asking them anything.” (35 years old, NGO 2 Call Center + Clinic, in-clinic procedural MR)*. Another client’s concern that providers would “make digs” at her for seeking MR care came to fruition,

They do the MR for you, but they still say things like that. ‘Why are you doing this? Did you not take any preventive measures in this regard?’ They are bound to say these kinds of things. And that is what happened, after I came here. *(22 years old, Maternity Hospital, in-clinic procedural MR)*

A call center client who was referred to NGO 2’s clinic described the doctor as “rude” and “irritable” and not willing to answer her questions about MRM,

Whenever I asked any questions, she [the doctor] got angry. Now it could be that she had to deal with a number of patients at the same time, and that was making her irritable. But as a patient, I have a lot of questions–this is a question of my life. *(25 years old, NGO 2 Call Center, in-clinic MRM)*

Two procedural MR clients reported feeling humiliated during their interactions specifically with reception staff during in-clinic care. For one client this was during the check-in process at the maternity hospital where she was scolded by the receptionist who said, *“Why do you come to us with all this bad news? Try to come to us with some good news.”* Another client at the NGO clinic was unclear about the processes of care within the clinic, including where to sit when. She said that the receptionist at this facility made her feel worse about her uncertainty by the way she spoke to her.

## Discussion

This study describes the expectations and experiences of MR clients from a range of service delivery settings in Bangladesh in order to better understand what elements of care delivery are important to clients and how they define high-quality MR care. Clients emphasized that positive interactions with providers rest heavily on how providers speak to them and the verbal encouragement providers give throughout care. This included speaking politely, explaining what to expect, and providing reassurance. When providers detailed each step of care and assured clients that they would be safe, it allowed clients to feel more confident in the care they received and even their decision to seek MR services. Findings from this study further reinforce the importance of client-provider communication and supportive care as key quality of care domains for person-centered MR care [18], especially among a client population like that in Bangladesh where many clients do not know what to expect during their care.

The desire for reassurance sought from their providers is likely connected to the lack of knowledge many had about MR prior to care. Clients held fears about what MR was and what would happen to them if they received MR care, most importantly concerns about infertility or damage to their uterus. This is consistent with existing literature on low levels of knowledge of MR among Bangladeshi women [6]. In some cases, misinformation on the safety of MR was perpetuated by MR providers. Clients reported receiving information about risks to their fertility both in printed materials and in conversations with providers during counseling sessions, specifically among participants who had more than one MR or who were nulliparous. Understanding how pervasive fear of future infertility is, both among clients and among some providers, points to opportunities for intervention to clarify the safety of MR in the community at large and at health facilities in Bangladesh. It is essential for providers to clarify information for clients when coming in for care rather than reinforce inaccurate information.

In addition to inaccurate information, clients reported experiences where providers and other staff attempted to counsel them out of their MR service. A previous qualitative study in Bangladesh explored women’s perceptions of MR care and found that providers were disrespectful at times by embarrassing the client [10]. Our findings add complexity to these findings because the same participants that described biased or coercive counselling also felt that they had received good care. This discordance may be related to internalized stigma or arriving at care with low expectations as was described by roughly half the sample of this study, and therefore not believing they deserved high-quality, non-biased care. It is common among abortion clients to express high satisfaction with services, regardless of experience, simply because they are no longer pregnant [23–27]. Additionally, biased counselling may have fulfilled expectations as clients worried they might be turned away for non-legal or medical reasons, such as parity, client age, spousal consent, and because the client was not married [11]. Similar to a validated scale used to measure person-centered abortion care in Kenya [28], this study demonstrates the importance that other staff throughout MR care, not just providers, also ensure that person-centered care domains of dignity and communication be practiced with clients. This is particularly relevant to in-facility MR care sites as clients must interact with staff at Reception and sometimes Triage before getting to care with the medical provider.

### Limitations

The qualitative nature of this study does not allow us to draw causal relationships between expectations and perceptions of care. Given the stigmatizing nature of abortion and MR, this relationship is worth exploring in future research in Bangladesh as well as other contexts. Our study was also not designed to compare experiences across sites of care, though there were patterns of treatment that emerged from in-facility care provision. It is also important to note that all of the participants in this study’s sample reported being married, which does not allow us to comprehensively report on the experiences and perspectives of unmarried women who seek MR in Bangladesh.

## Conclusion

Findings from this study identify a gap in what we know and understand about MR provider perspectives of the service they offer and of the clients they serve. It is clear that provider interaction with their clients weighs heavily on perceptions of quality and that providers present an important opportunity to improve accurate knowledge about the safety and effectiveness of MR, while also destigmatizing the procedure and the reasons for needing it. Clients rely heavily on the verbal and emotional support of their providers throughout care. Additional training with providers on accurate information provision is important, as well as ensuring that they continue to provide reassuring and transparent communication to clients throughout care.

### Electronic supplementary material

Below is the link to the electronic supplementary material.

Supplementary Material 1

Supplementary Material 2

## Data Availability

The datasets generated and/or analyzed during the current study are not publicly available due to privacy and confidentiality concerns but are available from the corresponding author on reasonable request. The interview guide is available from the corresponding author on reasonable request. Request to access the interview guide should be directed to sbaum@ibisreproductivehealth.org.

## Footnotes

1 We acknowledge that not all people who are capable of pregnancy and/or abortion identify as women. We use the term ‘women’ when referring to prior research that describes study subjects as such and we use the gender inclusive term ‘people’ whenever possible to acknowledge the diversity of people with uteri and the diversity of pregnancy and abortion experiences.

## Abbreviations

MR: Menstrual Regulation
MRM: Menstrual Regulation with Medication
MVA: Manual Vacuum Aspiration
NGO: Non-governmental organization
